# Flotation recovery of barite from high-density waste drilling fluid using β-cyclodextrin as a novel depressant and its mechanism

**DOI:** 10.1371/journal.pone.0298626

**Published:** 2024-03-14

**Authors:** Xiaoyu Li, Shuixiang Xie, Yu Xu, Yu Xia, Yuanpeng Cheng, Shanfa Tang, Duoqi Wang, Wen Ren, Mingdong Zhang, Wenyu Meng

**Affiliations:** 1 Department of Petroleum Engineering, Yangzte University, Wuhan, China; 2 Hubei Key Laboratory of Drilling and Production Engineering of Oil and Gas, Wuhan, China; 3 State Key Laboratory of Petroleum Pollution Control, Beijing, China; 4 CNPC Research Institute of Safety and Environmental Technology Co. Ltd, Beijing, China; Sivas Cumhuriyet University, TURKEY

## Abstract

High-density waste drilling fluid contains an abundance of recyclable weighting reagents, direct disposal can pollute the environment. In this paper, the primary mineral composition of a high-density waste drilling fluid from a well in the southwest oil and gas field was analyzed. This paper proposes β-cyclodextrin (β-CD) as a depressant for the recovery of barite from waste drilling fluid. The recovery process was investigated through inverse flotation experiments, and the mechanism was analyzed using zeta potential, contact angle analysis, and FTIR. The flotation experiments showed that under the SDS flotation system, when the pH was 9.0 and the amount of depressant β-CD was 900 g/t, the barite recovery and density reached the highest values, which were 87.41% and 4.042 g/cm^3^, respectively. Zeta potential experiments, contact angle analysis, and FTIR analysis indicate that β-CD adsorbed onto barite through enhancing the hydrophilicity of barite, electrostatic force adsorption, and strong adsorption, which could not be displayed by SDS through competitive adsorption. Furthermore, β-CD exhibited a selective inhibitory effect on barite and enabled reverse flotation. The mechanism model of the flotation separation process was established.

## 1. Introduction

Barite (BaSO_4_) is an essential non-metallic mineral that is widely used in oil and gas drilling, chemical engineering, metallurgy, and other industrial fields [1–3], particularly plays a vital role as a mud weighting reagent in oil and gas drilling [4–6]. As a major country in terms of barite reserves and consumption [7,8], long-standing issues with rough development and over-consumption, led to a composition of "poor, fine, mixed" and resulted in a shortage of high-quality barite. Simultaneously, a significant quantity of barite is transported from the well to the surface as a drilling mud weighting agent and the waste drilling fluid created in this process is considered hazardous waste with high-risk factors. Waste drilling fluid contains soluble inorganic salts, heavy metals, organic hydrocarbons, polymers, and other pollutants, which are characterized by high chromaticity, high COD, and high minerality. If not treated in a timely manner, serious environmental risks will be posed to surrounding soils, water sources, farmland and air when soaked by rainfall or washed away by rivers. This waste presents challenges in handling due to the toxicity and other associated problems. On the other hand, the recovery and reuse of barite can serve as a potential resource pool, offering an effective solution to alleviate the shortage of barite resources in China [9]. Furthermore, the approach aligns with environmental protection and promotes green production practices. Therefore, in the context of "Carbon Neutral, Carbon Peak," the reduction, harmlessness [10], and resource utilization of waste drilling fluids have become the goals of low-carbon sustainable development in the field of oil exploration [11].

A large amount of literature and field tests show that current treatment methods for waste drilling fluids can be broadly categorized into two groups: conventional methods and bioremediation. Conventional methods include curing treatment, incineration, and in-situ burial [12–14]. Hameedi [15] utilizes the MTC method (Mud to Cement) to incorporate chemical treatment agents into waste drilling fluids, which converts the waste drilling fluids into cementing materials. However, it necessitates the evaporation and drying of the liquids present in the drilling fluids. The latent heat of vaporization poses a significant challenge in terms of high energy consumption. Additionally, incineration and in-situ burial methods can lead to environmental pollution. On the other hand, bioremediation methods involve land cultivation and microbial treatment techniques [16,17]. Land cultivation method is to utilize the soil’s own purification characteristics to absorb the pollutants in the waste drilling fluids, and microbial treatment technology utilizes microorganisms to degrade the hazardous pollutants in the waste drilling fluids, but both of these methods have the shortcomings of long duration, difficult to achieve high biodegradation rates [18].

Therefore, several of the aforementioned methods have certain shortcomings in terms of resource utilization, resulting in the significant waste of valuable solid-phase resources, such as barite, found in waste drilling fluids. Thus, the development of high-efficiency and low-energy barite recovery technology is of great significance in the treatment of waste drilling fluids. Waste drilling fluid is a complex steady-state colloidal system that contains a significant amount of soluble salts [19–21], barite, clay, rock chips, and other minerals, which are prone to modification and have a high clay content [22]. Therefore, it is difficult to achieve effective separation and recovery of barite using only physical methods such as simple physical sedimentation and centrifugal separation. and it is necessary to further optimize and improve the existing treatment methods.

The technology used to recover barite from waste drilling fluids is adapted from mineral separation techniques used in the mining industry. The method of barite ore selection is employed to recover barite from waste drilling fluid. The primary techniques utilized in conventional mineral processing operations to separate ores are manual sorting, gravity concentration and flotation [23,24]. Among them, the manual sorting method is inexpensive but inefficient for beneficiation [25], the gravity concentration is suitable to be used in combination with other methods because achieving the desired effect by single use is difficult, and the flotation method is based on the difference in floatability between barite and other minerals, and the method of recovery is based on the addition of flotation chemicals to enhance the difference in surface properties of minerals [26–29]. Wang [30] utilized gellan gum as a depressant and investigated its action mechanism through zeta potential and FTIR analysis. The results revealed that gellan gum exhibited strong adsorption on the surface of barite, enabling the successful flotation separation of fluorite and barite. Deng [31] employed acidified water glass (AWG) as a depressant and sodium oleate (NaOL) as a collector, experimental findings demonstrated that AWG hindered the adsorption of NaOL on the surface of calcite, thereby increasing the difference in the mineral floatability of minerals and achieving the separation of barite from calcite.

β-CD is an environmentally friendly and harmless organic compound that can enhance the stability of drugs in the pharmaceutical industry. β-CD and its derivatives also have a certain effect on the treatment of heavy metals in water [32,33]. β-CD contains a large number of hydroxyl reactive functional groups and has the potential to be used as a green depressant in the flotation process. This is because its functional groups are prone to interact with the metal sites on the mineral surface. However, no studies have been found in the field of petroleum exploration and development on the application of β-CD in the flotation of waste drilling fluids with useful solid phases. Sodium dodecyl sulfate (SDS), as a collector, has excellent foaming properties, biodegradability, and stability over a wide pH range, SDS has been studied as a mineral flotation agent, and El‐Midany [34] compounded SDS with oleic acid for flotation, and obtained minerals with high recoveries and purity, thus SDS was used as a flotation collector for barite.

In this work, a new depressant β-CD with selective depressant was selected through flotation experiments based on detailed mineralogy research of high-density waste drilling fluid from shale gas wells in southwest oil and gas fields. The flotation experiment conditions were then optimized. The feasibility of the recovery reagent and process was verified through a full process closed-circuit test. Furthermore, the influence of β-CD on the mineral surface properties was explored through zeta potential measurements, contact angle experiments, and FTIR analysis. Finally, the mechanism model for the depressant β-CD in flotation separation was established. Therefore, the research results applied β-CD to the flotation of barite for the first time, which provides a feasible and economical process method for safe and optimal drilling, resource recovery, and utilization and green development in oil and gas fields, and also provides reference for practical applications.

## 2. Materials and methods

### 2.1. Minerals and reagents

High-density waste polysulfide drilling fluid was obtained from the centrifuge discharge port of a shale gas well in southwest oil and gas field, the density of drilling fluid is 2.150 g/cm^3^, the water content is 10.00%, and the barite content is 79.00%.

Dilute hydrochloric acid as pH adjuster, analytical-grade sodium dodecyl sulfate (SDS), and β-cyclodextrin (β-CD) were utilized as experimental reagents. Distilled water was used throughout the entire experiment.

### 2.2. Flotation experiment process

**Fig 1:**
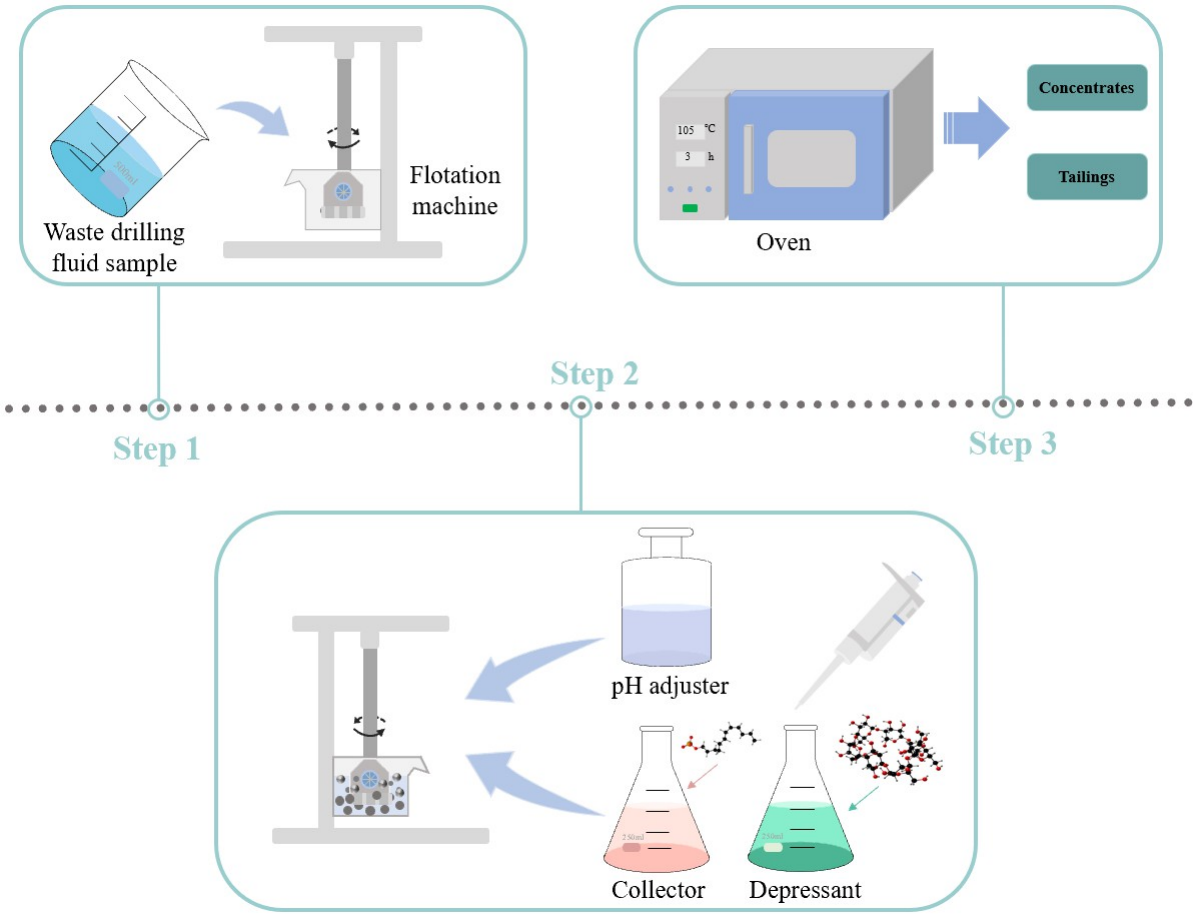
The schema of the flotation experiment.

Throughout the whole flotation process, an XFD_Ⅲ_ type 1.0L hanging tank flotation machine (Jiangxi Victor International Mining Equipment Company, Jiangxi, China) was used. A mass of 200 g was weighed, and the total volume of sample and reagent addition after dilution was controlled to 1.0 L. The mixture of sample and distilled water according to the set ratio was introduced into the flotation tank and stirred at 1600 rpm for 2 min. The pH of the pulp was adjusted with HCl, the reagents were added sequentially at 2 min intervals, the flotation lasted for 6 min, the solid slag adhering to the scraper plate was collected in the foam collection tank and the flotation tank was removed after flotation, and the product in the flotation tank was filtered, dried and weighed, and the recovery and density of barite were calculated. The flow of the flotation experiment is shown in Fig 1.

### 2.3. Closed-circuit floatation experiment

**Fig 2:**
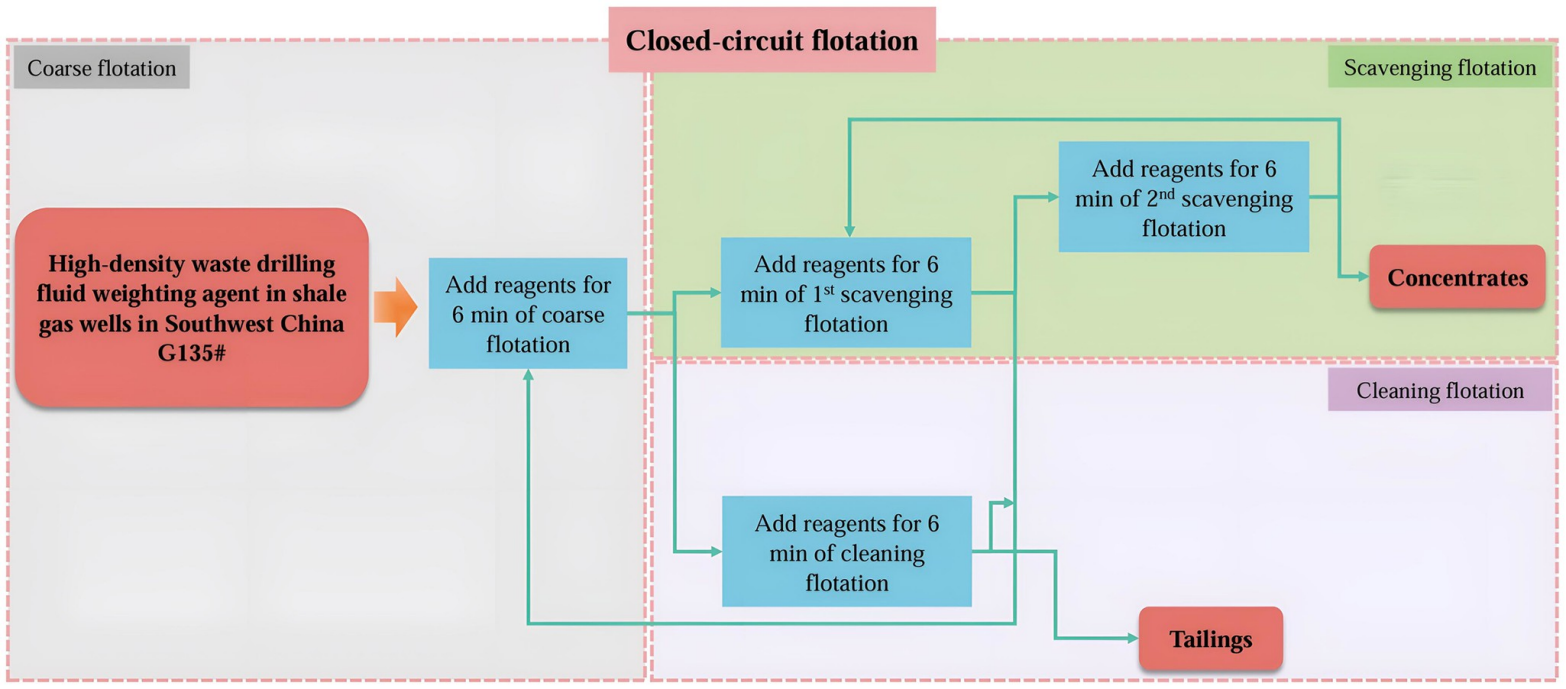
Closed-circuit flotation experiment flow.

The flotation experiment using the flotation machine continued based on the coarse flotation experimental conditions, conducting two scavenging flotations and one cleaning flotation for the waste drilling fluid sample. The flotation reagents chosen in the coarse flotation were added to the scavenging flotation and cleaning flotation, following the frequency and time intervals set during coarse flotation. After the flotation process, the concentrates and tailings were filtered and dried, the recovery and density were calculated. The closed-circuit flotation experiment flow is depicted in Fig 2.

### 2.4. XRD analysis

An X-Ray Diffractometer (dynamic 500, Anton Paar Shanghai Trading Co.) was used to detect mineral composition. An appropriate amount of high-density waste drilling fluid sample was dried in a drying box until it reached a solid state, and then fully ground with a grinder until it reached a particle size below 40 μm. 3 g of powder sample was weighed into the sample groove for measurement, and then pressed and compacted using a clean glass sheet. After that, the treated ore sample was analyzed and tested on an X-ray diffractometer.

### 2.5. Particle size analysis

The Malvern laser particle sizer (Mastersizer 2000, Shanghai Spectrum Instrument Systems Co.) and high-power CNC ultrasonic cleaner were used for particle size analysis. 2 g of waste drilling fluid sample was added to 20 ml of deionized water in a conical flask. Ultrasonic shaking was carried out for 10 min to ensure proper dispersion. After through mixing, the particle size of the sample was analyzed using a Malvern laser particle sizer.

### 2.6. Zeta potential measurements

A zeta potential meter (Nanoplus, Micromeritics Shanghai Instruments Co.) was used to determine the zeta potential of barite and quartz after reaction with different flotation reagents. 50 mg barite and quartz samples with a particle size of -5 μm were mixed with 80 ml KNO_3_ electrolyte solution, mixed evenly at a certain pH, and their suspension was taken for zeta potential measurement.

### 2.7. Contact angle experiments

Mineral surface wettability can be visualized by the size of the contact angle. The contact angle of the minerals was measured using a contact angle meter (Data Physics-OCA20, Germany DataPhysics Instruments Co.). High-purity barite and quartz mineral blocks with smooth and flat surfaces were carefully chosen, and then polished using a polishing machine and the mineral surfaces were subsequently cleaned and dried using ultrasonic technology. The contact angle of the mineral sample after reacting with flotation chemicals at the required concentration was measured using the solid drop method. The samples were sequentially immersed in the desired flotation reagents for a fixed immersion time of 3 min, and the mineral surface was washed with deionized water and dried. Afterward, the measurements were repeated three times, and the average value was calculated.

### 2.8. FTIR analysis

FTIR analysis (INVENIO, Bruker (Beijing) Technology Co.) was conducted using an FTIR spectrometer. The infrared spectra of the samples were determined using the potassium bromide pressing method. 10 mg of the mineral samples to be tested were mixed with the appropriate concentration of flotation reagent. The mixture was stirred for 30 min with a magnetic stirrer, filtered, and dried. Then 1 mg of the samples was weighed and pressed with 100 mg of potassium bromide in a mortar and pestle. The resulting mixture was pressed into tablets and subjected to the FTIR test [35].

## 3. Results and discussion

### 3.1. Sample analysis results

#### 3.1.1. XRD results

The X-ray diffraction analyzer was used to characterize the high-density waste polysulfide drilling fluid sample G135# from the southwest oil and gas field. The analytical results are presented in Table 1.

**Table 1:** Mineral composition and content of high-density waste drilling fluid sample G135# from Southwest oil and gas field.

Sample number	Mineral content(%)			
	Barite	Quartz	Calcite	Clay minerals
G135#	79.00	11.00	7.00	3.00

As shown in Table 1, the primary solid-phase composition of minerals in the high-density waste polymer-sulfonate drilling fluid in the southwest oil and gas field includes barite (BaSO_4_), quartz (SiO_2_), calcite (CaCO_3_), and clay minerals. These minerals account for 79.00%, 11.00%, 7.00%, and 3.00%, respectively. Among these minerals, barite has the highest composition, accounting for 79.00% of the mineral fraction. and the low-density mineral impurities contained the sample are quartz, calcite, and clay minerals. Therefore, the focus of the barite beneficiation for this high-density waste drilling fluid is to remove the aforementioned low-density mineral impurities through flotation and obtain high-quality barite.

#### 3.1.2. Particle size analysis results

**Fig 3:**
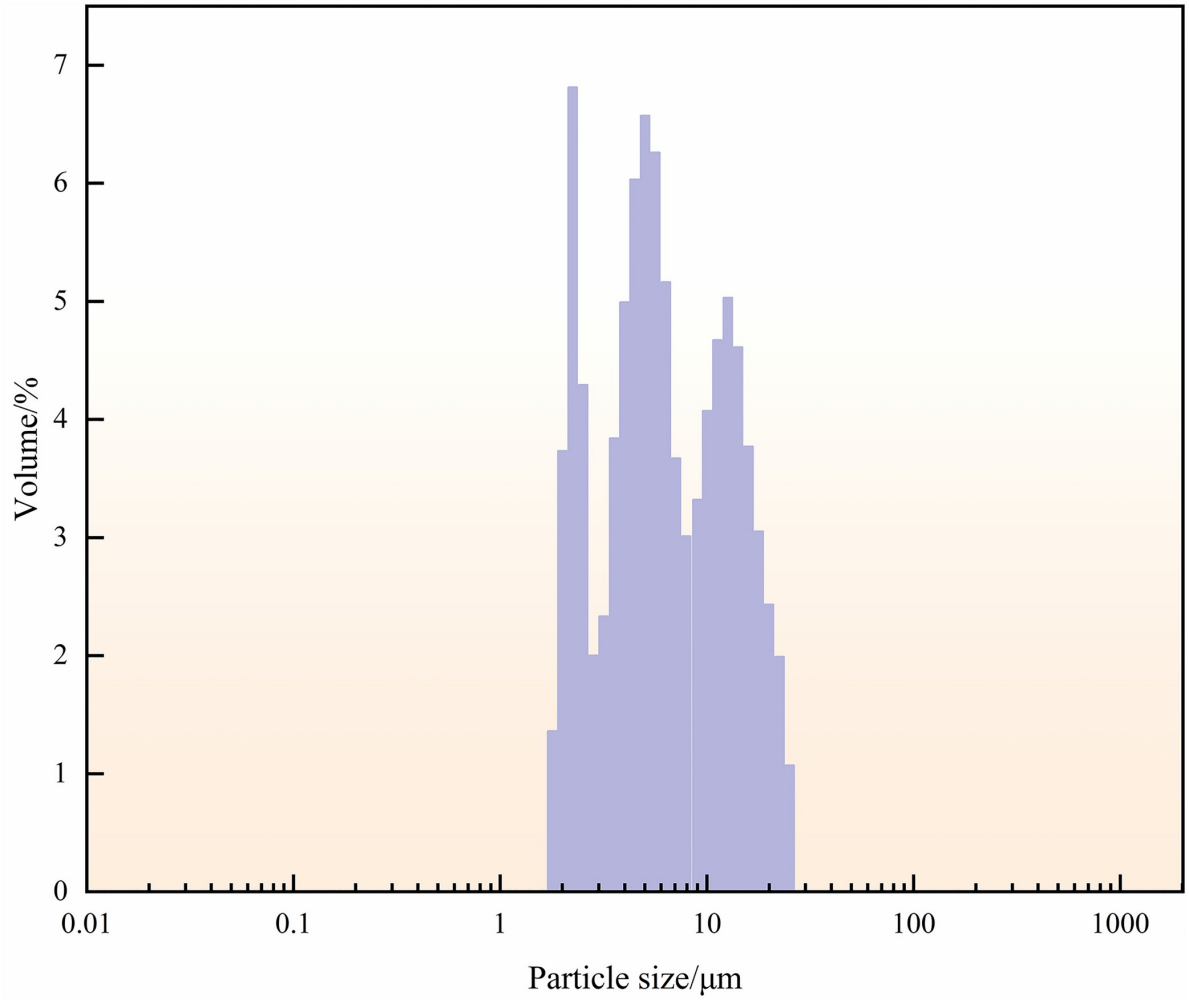
Particle size distribution of high-density waste polysulfonate drilling fluid in the Southwest oil and gas field.

Particle size analysis was conducted on samples of high-density waste drilling fluid, and the results are presented in Table 2 and Fig 3.

**Table 2:** Particle size analysis results of high-density waste polysulfonate drilling fluid samples from Southwest oil and gas fields.

Sample	d (0.1), μm	d (0.5), μm	d (0.9), μm
Raw pulp (Ultrasonication)	2.169	5.845	18.639
Raw pulp (Not ultrasonically treated)	2.134	5.208	16.391

As shown in Table 2, the particle size of the high-density waste drilling fluid remained relatively stable before and after ultrasound treatment, with a median change of less than 1 μm, which indicates that the particles generally become finer-grained. As shown in Fig 3, the particle size distribution of this high-density waste drilling fluid exhibits a small peak, with the majority of fine particles ranging from 1–10 μm. Therefore, the particle size of waste drilling fluid mineral has minimal effect on the experimental results, and there is no need for ultrasonic treatment in the subsequent experiments.

### 3.2. Flotation experiment results

#### 3.2.1. Collector dosage experiment results

**Fig 4:**
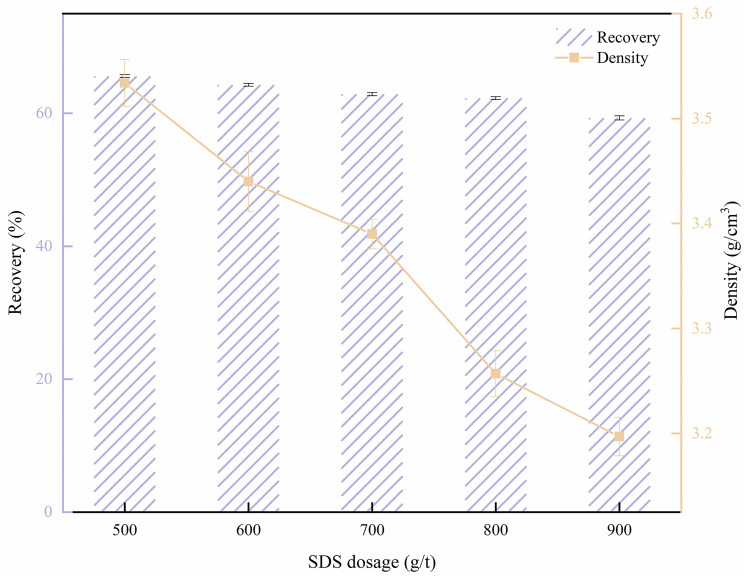
Effect of sodium dodecyl sulfate (SDS) on barite flotation.

SDS is an anionic surfactant commonly used as a collector in flotation. In this study, SDS was employed as a collector to investigate its impact on barite flotation [36]. The flotation experiments were conducted before and after β-CD treatment to investigate the impact of collector and depressant dosage on the flotation behavior of barite. The SDS dosage experiment results are depicted in Fig 4.

As shown in Fig 4, the increase in SDS dosage led to a decrease in barite recovery and concentrate density [37,38]. It is analyzed that SDS, as a surfactant, exhibits both collecting and foaming properties [39]. With the higher SDS dosage, the foaming effect becomes significant. However, due to the lack of selectivity, this also results in together flotation of barite and low-density solid phases, leading to a decrease in concentrate yield. This decrease in barite recovery and the subsequent reduction in concentrate density present a challenge to the flotation separation of barite from low-density solid phases [40]. Therefore, the use of depressant is crucial for the effective flotation separation of barite [41,42].

#### 3.2.2. Depressant β-cyclodextrin(β-CD) experiment results

**Fig 5:**
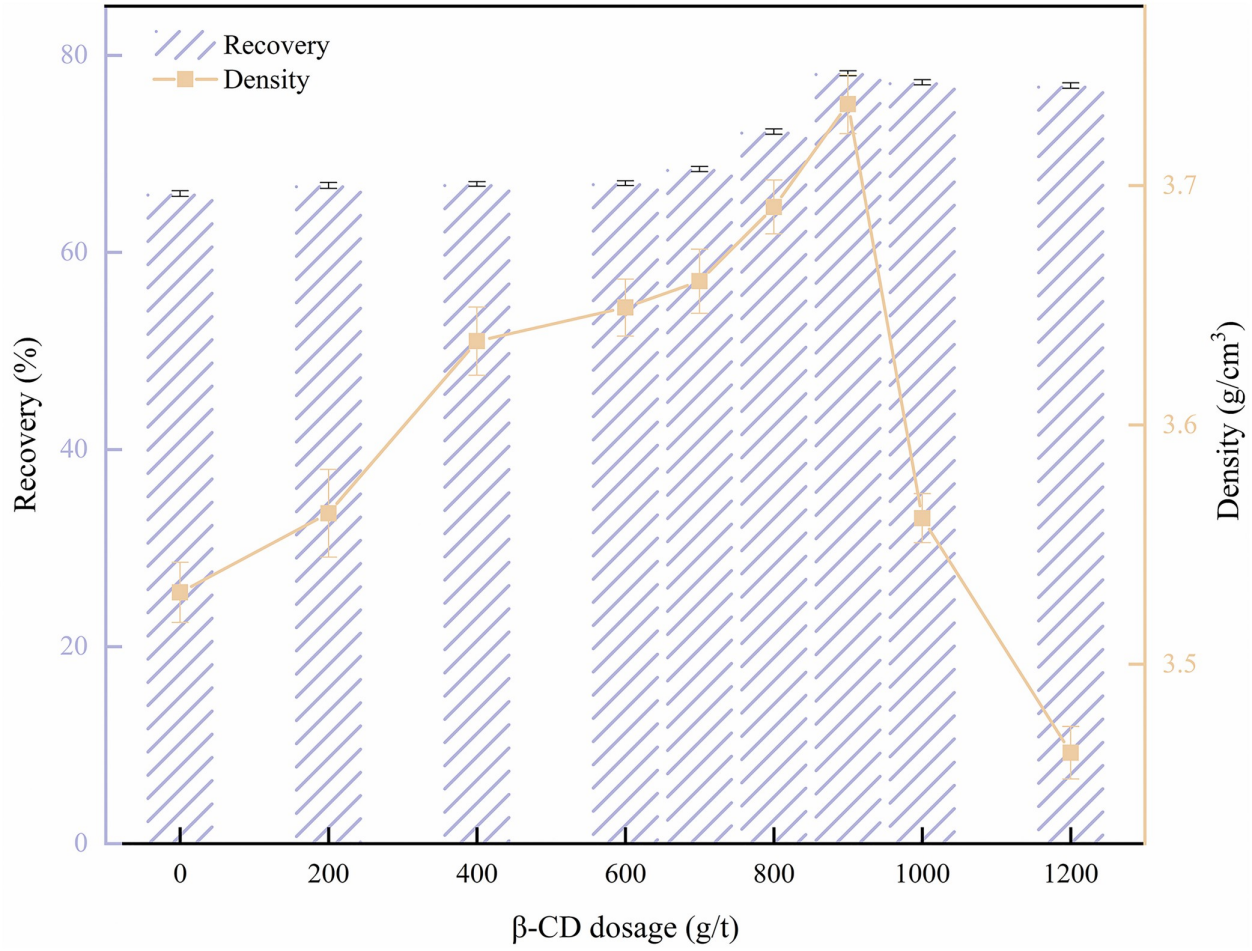
Effect of β-cyclodextrin (β-CD) on barite flotation.

Fig 5 illustrates the correlation among β-CD dosage and barite recovery and concentrate density. As depicted in Fig 5, the recovery of barite and concentrate density gradually increased with the rise in β-CD dosage, reaching the peak at 900 g/t. At this point, the effect was optimal, with the recovery and concentrate density at 78.19% and 3.734 g/cm^3^, respectively. However, further increase in β-CD dosage led to a decline in the recovery of barite and concentrate density. The trend of first increase and then decrease may be attributed to the initial increase in β-D dosage when adding the depressant. At the onset, β-CD exhibited an inhibitory effect on barite, with the most significant depression observed at a dosage of 900g/t. This resulted in a large amount of barite being retained in the concentrate, leading to an increase in the barite content and the density of the concentrate. Furthermore, as the β-CD dosage continued to increase, the excess depressant adsorbed on the surface of other minerals, leading to the coexistence of barite and low-density solid phase in the concentrate, which resulted in a decrease in concentrate density [43].

#### 3.2.3. Effect of pH on flotation results

**Fig 6:**
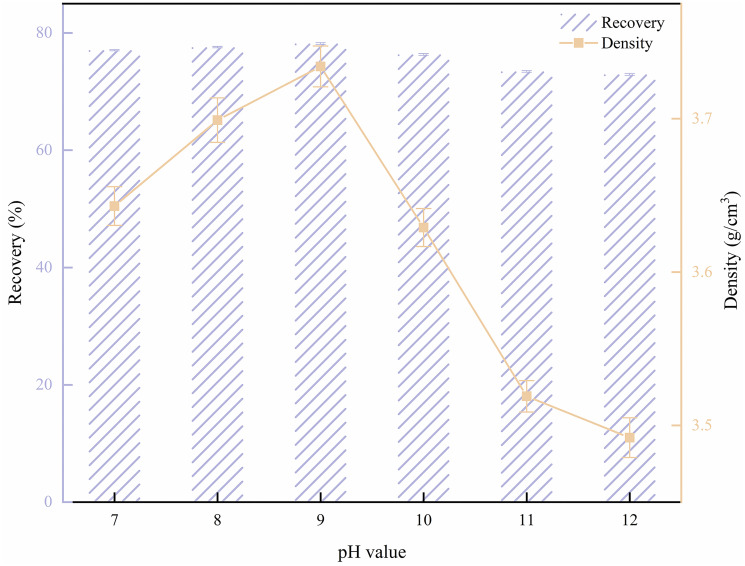
Effect of pH on barite flotation.

Under the experimental conditions of 500 g/t of SDS as the collector and 900 g/t of β-CD as the depressant, the pH of the pulp in the flotation process was optimized. Dilute hydrochloric acid (HCl) was used as the pH adjusting reagent. The results of the pH effect on the flotation behavior of barite are illustrated in Fig 6. As depicted in Fig 6, the pH of the pulp has a significant impact on the barite recovery and concentrate density. The barite recovery and concentrate density exhibit a pattern of initially increasing and then decreasing with the rise of pH value. When the pH of the pulp is lower than 9.0, the recovery and concentrate density increase with the rise of pH, and the optimal recovery and density are achieved at a pH value of 9.0. However, with the increase of pH value, the increase in recovery and density actually decreased instead. This phenomenon may be attributed to the increased presence of OH^-^ in a highly alkaline environment, causing more OH^-^ to be adsorbed on the surface of the mineral particles. This, in turn, hinders the adsorption of the flotation reagents and results in the entrainment of low-density solid phase in the concentrate, ultimately leading to reduced recovery and density.

#### 3.2.4 Full process closed circuit flotation experiment results

Closed-circuit flotation experiments were conducted on barite according to the process parameters and reagents determined in the previous conditional experiments. The HCl was used as a pH adjuster, β-CD was used as a depressant, and SDS was used as a collector in the experiment. The experiment results were shown in Table 3.

**Table 3:** The results of the closed-circuit flotation experiment.

Product name	Productivity, %	BaSO_4_, %	Recovery	Density, g/cm^3^
Concentrates	74.19	93.07	87.41	4.042
Tailings	25.81	38.53	12.59	3.368
Raw mineral	100.00	79.00	100.00	3.612

The results of the closed-circuit flotation experiments are presented in Table 3. It can be observed that after one coarse flotation, two scavenging flotations, and one cleaning flotation, the recovery of barite reached 87.41%. The barite content in the concentrate was as high as 93.07%, and the density of the concentrate was 4.042 g/cm^3^, which represents a significant increase in barite recovery and concentrate density compared to the results of the coarse experiments alone. Following the closed-circuit flotation experiment, the barite content in the tailings was effectively reduced, leading to a substantial retention of barite in the concentrate and a reduction in barite loss.

### 3.3. Zeta potential results

**Fig 7:**
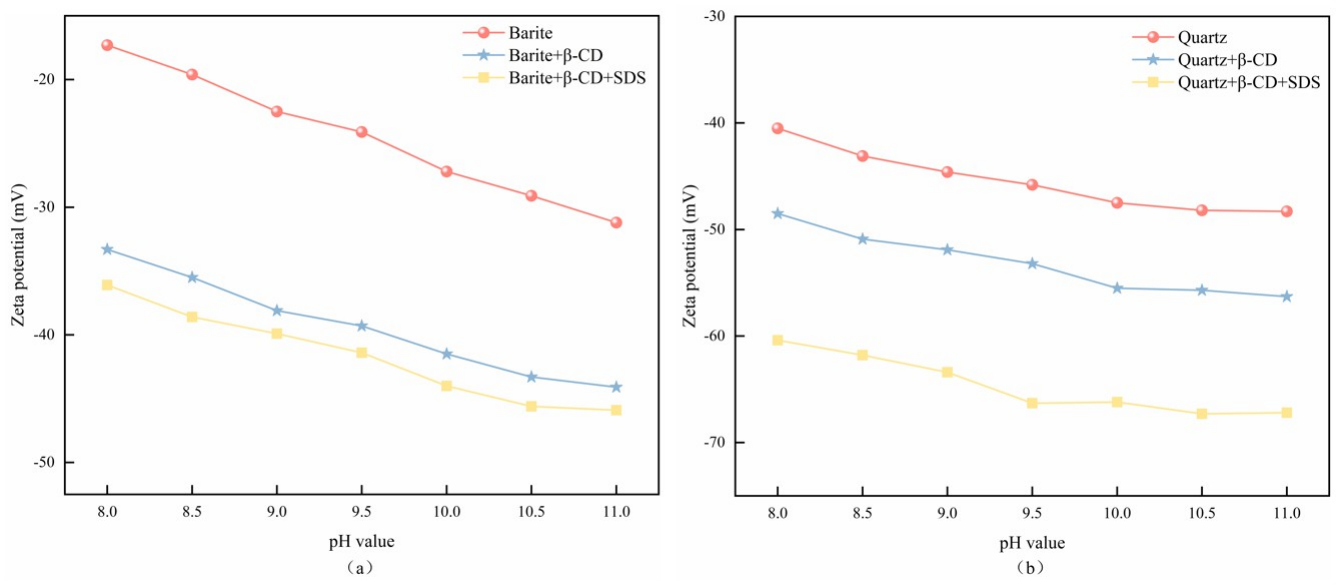
Effect of reagent adding on the zeta potential of barite and quartz.

The zeta potential is a characterization of the surface charge of minerals and provides insight into the adsorption behavior of flotation reagents on the mineral surface. The zeta potential of barite and quartz as a function of pulp pH under different treatment conditions is depicted in Fig 7. The zeta potentials of barite and quartz are negative at pH 8.0–11.0, which is consistent with previous study [44]. As depicted in Fig 7, pH value exerts a significant influence on the zeta potential of barite and quartz, consistently showing a decreasing trend with increasing pH across various treatment conditions. In the presence of the depressant β-CD, the zeta potential of both barite and quartz decreased significantly in the studied pH range, reaching values well below 0. Therefore, β-CD was found to be adsorbed on the surface of the minerals. For barite, the difference in the zeta potentials was maintained 12.9–16.0 mv with the addition of β-CD as compared to the absence of β-CD. At pH 9.0, the addition of β -CD resulted in a decrease of 15.6 mV in the zeta potential of barite. Fig 7(b) illustrates a negative shift in the quartz zeta potential with the addition of β-CD compared to the pure mineral, with a decrease in the zeta potential by 7.8 mV at the pH of 9.0. These results indicate different β-CD surface adsorption behaviors for barite and quartz. The zeta potential of quartz was further decreased by the continued addition of SDS, which was attributed to the fact that SDS acts as an anionic collector, and its molecules in aqueous solution form negative charges over a wide pH range. However, there was almost no effect on the zeta potential of barite after treatment with β-CD. This result indicates that SDS could still be adsorbed on the surface of quartz after β-CD treatment, while the adsorption on the surface of barite was completely blocked. These results show that β-CD effectively prevents the adsorption of SDS on the surface of barite.

### 3.4. Surface wettability results

**Fig 8:**
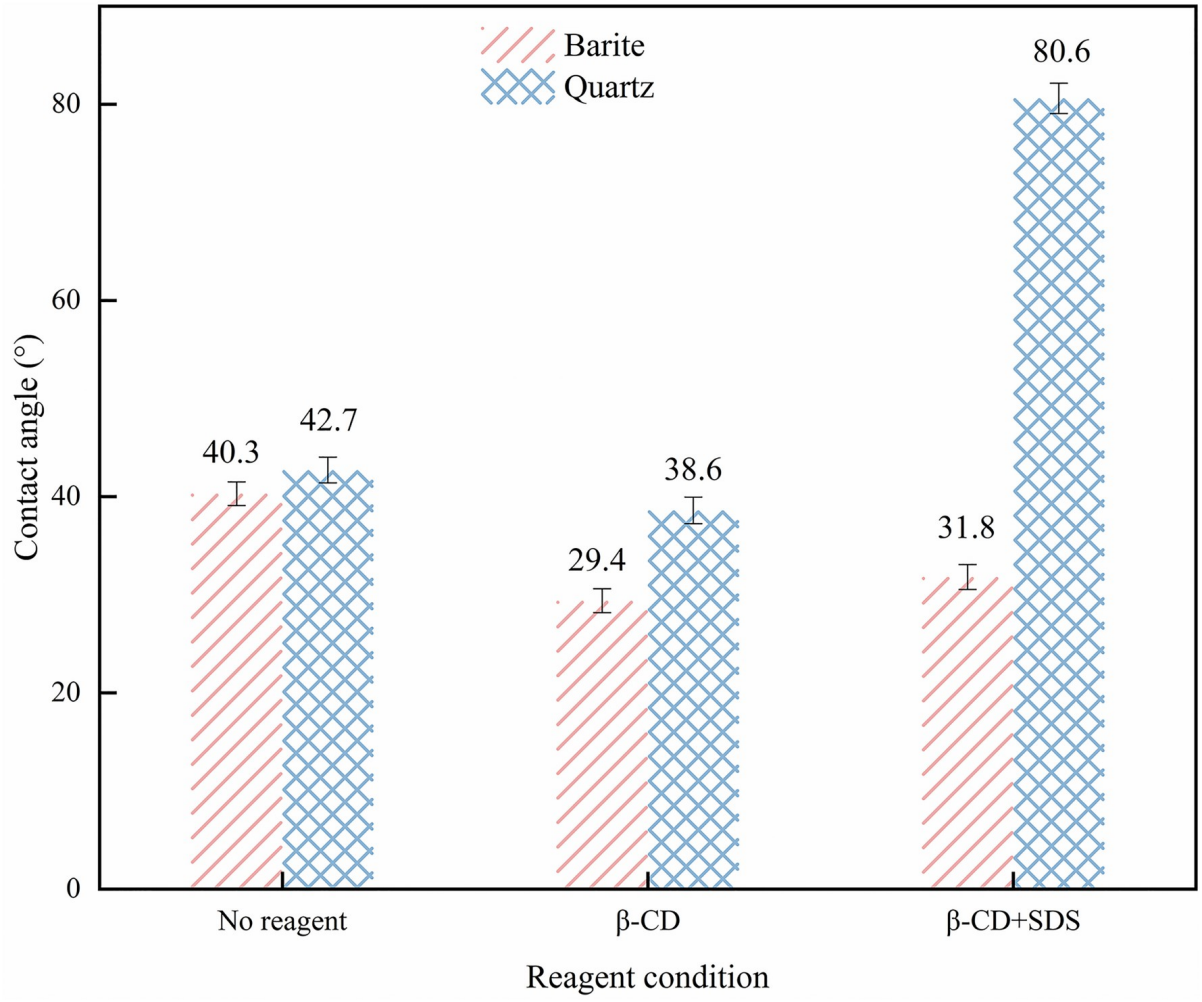
Contact angles of barite and quartz with different reagents.

Contact angle measurements are an important method for investigating mineral flotation properties [45]. The smaller the contact angle, the more hydrophilic the mineral surface is, and the more difficult it is to float the mineral [46]. The effects of the depressant β-CD and the collector SDS on the surface contact angle of barite and quartz are shown in Fig 8. The contact angles of barite and quartz were 40.3° and 42.7°, respectively, similar to previous research [47]. After the treatment with β-CD, the contact angles decreased to 29.4° and 38.6°, which indicates that the addition of β-CD significantly decreased the contact angles of the two minerals, therefore, increasing their hydrophilicity. The results demonstrated that β-CD can enhance the hydrophilicity of barite and quartz in the absence of the collector. When SDS was added and followed by β-CD, the contact angle of barite increased to 31.8°. indicating that the addition of the collector did not prevent the adsorption of the depressant on the barite surface, thus enhancing its hydrophilicity. This results in more barite remaining in the concentrate, which may account for the high barite recovery and concentrate density in this case. And The contact angle of the SDS-treated quartz increased significantly to 80.6°, indicating that the addition of β-CD did not affect the subsequent adsorption of SDS. The results demonstrated that the pre-treatment of β-CD had slightly affected the wettability of quartz surface and the subsequent SDS adsorption, while β-CD had a great effect on barite, enhancing its hydrophilicity and hindering the adsorption of SDS on its surface.

### 3.5 FTIR results

**Fig 9:**
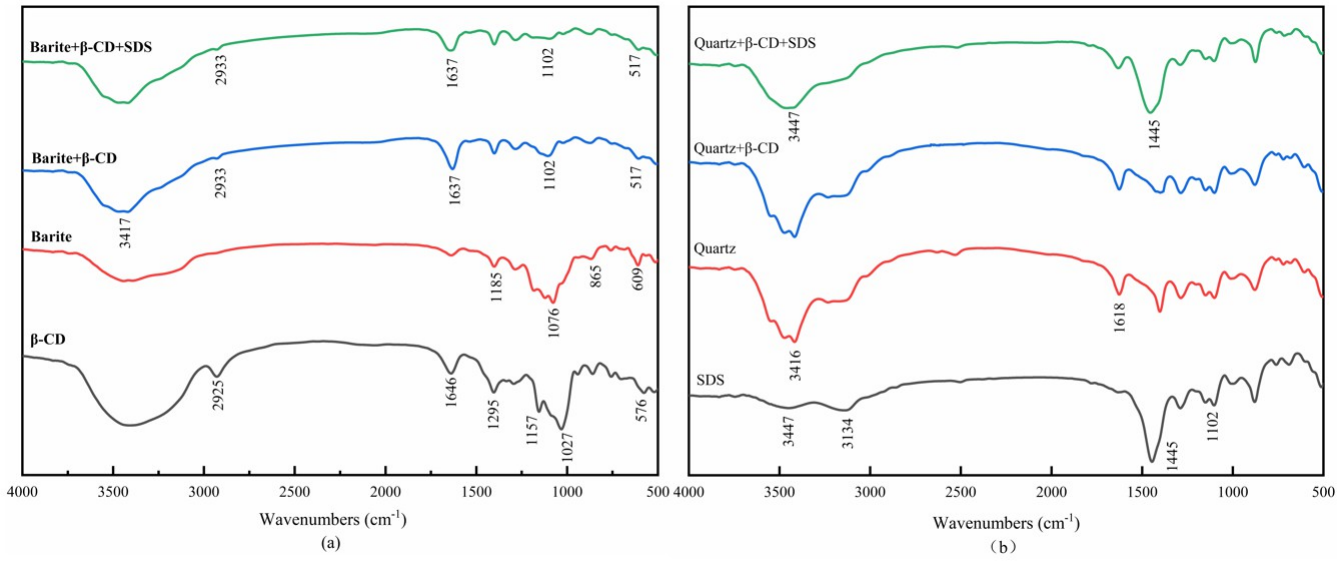
FTIR spectra of β-CD and untreated/treated barite and quartz.

FTIR testing is a widely used and effective method for studying exploring the adsorption mechanism of flotation chemicals on mineral surfaces [47]. If β-CD is strongly adsorbed on the surface of barite or low-density solid phase, on the other hand, if the adsorption of β-CD on the surface of the minerals is weak, there is no shift observed [48,49]. It can be seen from Fig 9(a) that the characteristic absorption peaks of barite are at 1185 cm^-1^, 1076 cm^-1^, 865 cm^-1^, and 609 cm^-1^. The peaks at 1185 cm^-1^ and 1076 cm^-1^ are stronger symmetric telescopic vibrational absorption peaks induced by SO_4_^2^-, the peak at 865 cm^-1^ is an asymmetric telescopic vibrational absorption peak induced by SO_4_^2-^, and the peak at 609 cm^-1^ is an in-plane bending vibrational absorption peak [50,51].

To understand the adsorption mechanism of β-CD on barite and low-density solid phase, FTIR was used to investigate the preadsorption of β-CD on minerals, and the results are presented in Fig 9(a). In the FTIR of β-CD, the peak at 2925 cm^-1^ corresponds to the stretching vibration of C-H bond, while the peaks at 1646 cm^-1^ and 1295 cm^-1^ are attributed to the bending vibration of -OH on the glucopyranose ring in the β-CD molecule, the characteristic peaks at 1157 cm^-1^, 1120 cm^-1^, and 1027 cm^-1^ indicate the C-O-C and C-O stretching vibrations, while the peak at 576 cm^-1^ represents the molecular backbone vibration of β-CD [52–55]. The FTIR of SDS is depicted in Fig 9(b), the peaks at 3447 cm^-1^ and 3134 cm^-1^ are the asymmetric and symmetric telescopic vibrational absorption peaks of C-H in the alkyl chain, respectively, the bending vibrational peak of C-H at 1445 cm^-1^ and the peak at 1102 cm^-1^ is the asymmetric telescopic vibrational peak of C-O-S [56,57]. Fig 9(a) depicts the infrared spectra of barite treated with various flotation chemicals, when treated with β-CD alone, the peaks of and C-H and -OH were detected at 3417 cm^-1^ and 1637 cm^-1^, respectively, at which time the characteristic peaks of C-O-C and C-C were clearly shifted to 1102 cm^-1^, indicating that β-CD was strongly adsorbed on the barite surface, while β-CD chemisorbed on the barite surface, and no new characteristic peaks appeared after further addition of the collector SDS, indicating that the β-CD pretreatment had a greater effect on the subsequent adsorption of SDS on the barite surface, preventing the adsorption of SDS on the barite surface. The results of the infrared spectra of quartz before and after pharmaceutical treatment are shown in Fig 9(b). In the FTIR spectrum of quartz, the characteristic peaks located at 3416 cm^-1^ and 1618 cm^-1^ correspond to the stretching and bending vibrations of -OH (Si-OH) [58], respectively. The characteristic peaks distributed in the range of 400 cm^-1^ to 1200 cm^-1^ represent the vibrational peaks of Si-O bonds. When the quartz was treated only with β-CD alone, there was no obvious change in the peaks compared to the FTIR spectra of the quartz, and no new peaks appeared, indicating that the adsorption of β-CD on the surface of the quartz was weak. However, after treating the quartz sequentially with β-CD and SDS, the characteristic peaks of SDS still appeared at 3447 cm^-1^ and 1445 cm^-1^, which proves that adsorption of SDS on the quartz had occurred. Therefore, the addition of β-CD could not prevent the subsequent adsorption of SDS on the quartz.

Thus, FTIR analysis revealed that β-CD weakly adsorbed onto the quartz and had little effect on the subsequent adsorption of SDS [59,60], whereas β-CD strongly adsorbed onto the barite surface and prevented further SDS adsorption on barite, which is consistent with the flotation results.

### 3.6. Possible depression mechanism and adsorption model

**Fig 10:**
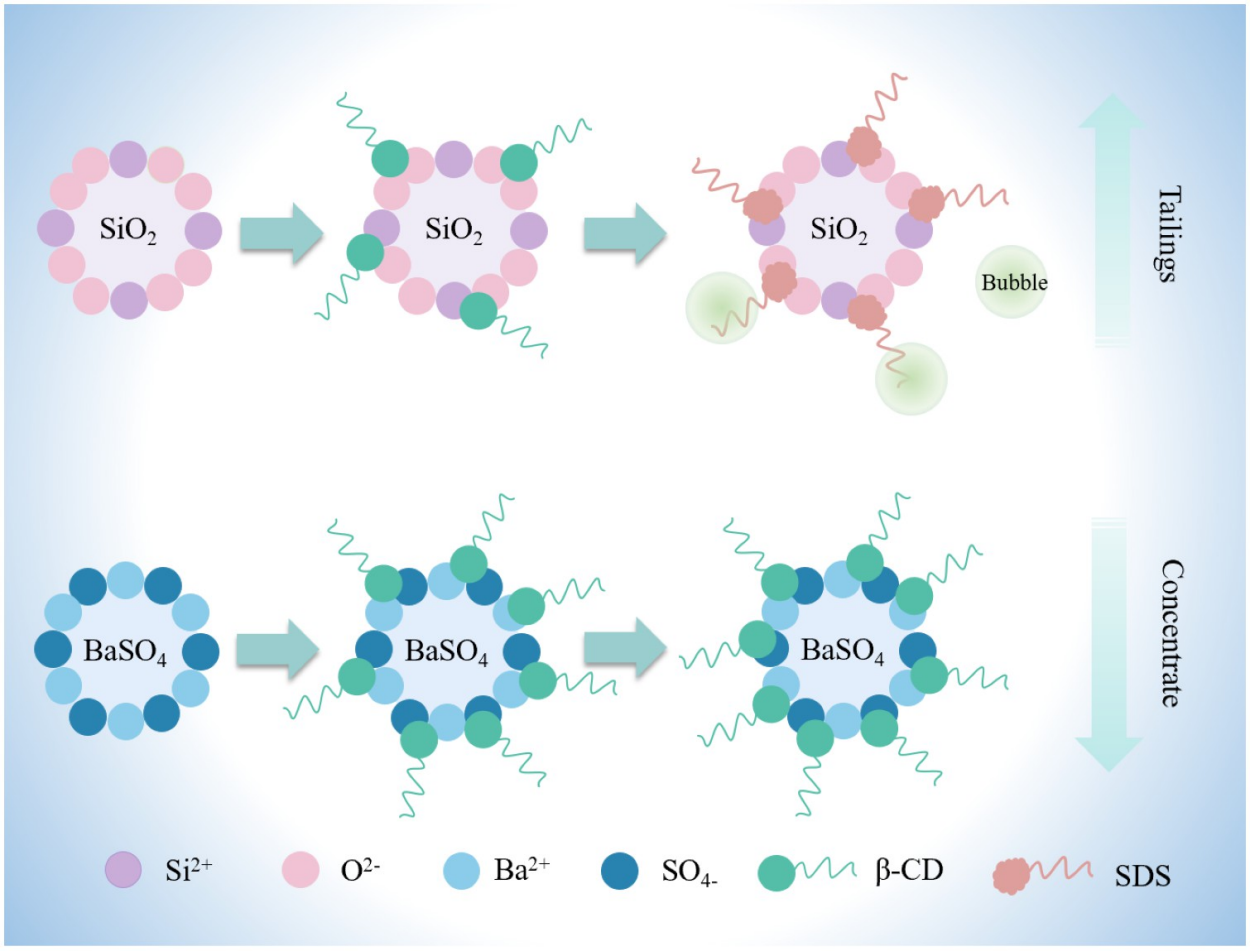
Proposed model for the adsorption of β-CD and SDS on the barite and low-density solid phase surfaces.

β-CD contains a large number of hydrophilic -OH groups, which are highly stable in alkaline environments. Therefore, it readily forms chelates with multivalent metal ion sites on mineral surfaces. However, the differences in crystal structure and surface charge between barite and quartz affect the adsorption effect of β-CD. Zeta potential and FTIR results indicate that β-CD adsorption occurs with varying strengths on barite and quartz. The adsorption of β-CD on the quartz surface may be determined by weak physical adsorption, such as electrostatic force and van der Waals force. This type of adsorption shows weak affinity and cannot prevent the subsequent adsorption of SDS on the surface. The hydrophobic groups present in SDS adsorb on the quartz surface, causing it to exhibit hydrophobicity, which facilitates froth flotation. β-CD exhibits stronger adsorption depression on barite compared to quartz. The adsorption of β-CD on the surface of barite involves stronger chemical adsorption due to the hydrophilicity of the outer edge of β-CD, which enhances the hydrophilicity of barite in the pulp and hinders the subsequent adsorption of SDS on the surface of barite, thereby inhibiting barite and allowing a large amount of barite to remain in the concentrates. This process enables the recovery of barite and an increase in density. The adsorption model is shown in Fig 10.

## 4. Conclusion

Through detailed process mineralogical analysis of the substances contained in the high-density waste polysulfonate drilling fluid sample from a shale gas well in the southwest oil and gas field, it is learned that the waste drilling fluid sample contains low-density mineral impurities, mainly quartz, calcite, and some clay components, and the barite grade is high at 79.00%, and the overall sample size range is 1–10 μm with a fine particle size. Through flotation experiments, β-CD is used as screening depressant, and the optimal dosage is 900 g/t. According to the test of full process closed-circuit flotation experiment, the optimal process parameters are identified as "one coarse flotation, two scavenging flotations and one cleaning flotation", which result in a grade of 93.07%, a recovery of 87.29% and density of 4.042 g/cm^3^ of BaSO_4_. Zeta potential experiments show that in the presence of the collector SDS, the depressant β-CD can be adsorbed on the surface of barite by electrostatic, reducing the floatability of barite, and this adsorption is stronger than that of quartz, and subsequently affects the adsorption of SDS on barite. The contact angle measurement experiments illustrate that β-CD impedes the adsorption of SDS, while adsorption on the surface of barite expands the hydrophilicity of barite, FTIR analysis reveal that the adsorption of β-CD on the barite surface is attributed to strong adsorption and cannot be replaced by SDS through competitive adsorption, thus achieving depression and counter-flotation. A mechanism model of flotation separation of barite with SDS as a collector and β-CD as a depressant is established, moreover, the possible reasons for the selective adsorption of β-CD on the barite surface are as follows:(a) β-CD requires less electrostatic repulsion to overcome when adsorbed on the barite surface; (b) the adsorption of β-CD on the barite surface may be due to a combination of electrostatic forces and strong adsorption.
